# Physiotherapeutic Approach for Volar Barton Fracture Left Side Without Neurological Deficits: A Case Report

**DOI:** 10.7759/cureus.45363

**Published:** 2023-09-16

**Authors:** Shraddha S Kochar, Snehal Samal

**Affiliations:** 1 Neurophysiotherapy, Ravi Nair Physiotherapy College, Datta Meghe Institute of Higher Education and Research, Wardha, IND

**Keywords:** case report, physiotherapy, rehabilitation, distal radius fracture, volar barton fracture

## Abstract

Distal radius bone fracture termed a volar Barton fracture takes place when the ventral portion of the bone is displaced. This fracture happens obliquely within the joint itself. This is the case report of a 52-year-old female, who met with a road traffic accident and injured her left wrist. After the necessary investigation, she was diagnosed with a volar Barton fracture of the left wrist without any neurological deficits. After this, she was managed with open reduction and internal fixation with plate osteosynthesis. Postoperatively, the patient complained of pain around the left wrist joint and there was also restriction in the movements of the left wrist, which caused it to be difficult to perform everyday tasks. We report that following surgery, physical therapy intervention significantly reduced the intensity of pain, improved the range of motion, and strength of the muscle, and remarkable progress in the patient's functional independence. An Upper Extremity Functional Index was used as an outcome measure.

## Introduction

An intra-articular fracture influencing the volar or dorsal lip describes Barton's fracture, a radiocarpal joint fracture-dislocation [1]. In clinical settings, the lower-end radius fracture contributes to 17% of all fractures and 75% of all forearm fractures and becomes one of the most typical fractures of the upper limb [2]. Both genders are more exposed to such injuries during their fifth and sixth decades, however, the incidence is maximum among women during their fourth decade, which is going through menopause age (peri-menopausal) [3]. Volar Barton distal radius fractures are referred to as oblique fractures that affect the volar articular edge of the distal radius involving proximal and volar displacement of the carpus as it corresponds to the volar fracture fragment [4]. These fractures are classified as unstable, and there are many different treatments that can be found in the research, namely close reduction and plaster application, external fixation, pin fixation, and plaster and plating with or without the grafting of bone [5]. It has been established that applying a volar plate for internal fixation possibly unstable distal radial fractures enhances the likelihood of a painless union compared to more traditional treatment approaches [6]. The functional outcome is further enhanced through early post-operative mobilization [7]. In the beginning, closed reduction was implemented for treating volar Barton fractures, but at present, the approach has shifted to open reduction internal fixation (ORIF) in conjunction with the earliest possible mobilization to treat these periarticular fractures [8]. The most important objectives in therapy for a Volar Barton fracture include fracture stability, regaining the range of motion (ROM), and avoiding the possibility of sequelae [9].

## Case presentation

A 52-year-old woman from a rural area who has right-hand dominance suffered injuries in a road traffic accident (a fall from a scooter) and sustained injury to her left wrist and some minor abrasions around the forearm and knee joint. She was aware and alert without a history of head trauma when brought to AVBRH, Sawangi, by car. After conferring with an orthopedic physician, tests like x-rays and CT scans suggested a left-side volar Barton fracture without any neurological damage. The patient underwent surgical treatment, ORIF with plate osteosynthesis for distal end radius fracture left side operated on January 16, 2023. Following the procedure on the next day, treatments for physiotherapy were initiated. The patient's primary complaints following surgery were pain around the left wrist, which is gradual in onset, stabbing in nature with intensity 7/10 on movement and 2/10 on rest on the Numerical Pain Rating Scale (NPRS), and restriction of movement of the left wrist. She has a known case of hypothyroidism and hypertension currently on medications for two years and diabetes mellitus for five years not on medications.

Clinical findings

Before performing the examination, permission was taken from the patient. She was assessed physically while seated, and her vital signs were afebrile, 130/90 mmHg blood pressure, and 84 beats/minute heart rate. On inspection, the patient’s left shoulder was abducted slightly, flexed, and internally rotated, the elbow was in 90 degrees of flexion and the wrist was approximately in 5 degrees of flexion. There was the presence of abrasion around the forearm and knee anteriorly, bony deformity, and diffuse swelling present around the left wrist. On palpation, local temperature was raised, diffuse tenderness around the wrist joint with grade 2, abrasion of 2x2 cm were present around the left forearm and 4x4 cm over the left knee, radial artery was palpable. All sensations and reflexes were present and responsive following neurological evaluation. Table 1 shows the ROM on the first day and Table 2 shows manual muscle testing on the first day.

**Table 1:** On the first day of rehabilitation, the range of motion of left side joint assessed.

Joint	Active Range of Motion (in degree)	Passive Range of Motion (in degree)
Elbow Flexion	0-145	0-150
Forearm Pronation	0-76	0-80
Supination	0-75	0-80
Wrist Flexion	0-10	0-15
Extension	0-5	0-8
Radial deviation	0-2	0-5
Ulnar deviation	0-5	0-8

**Table 2:** On the first day of physical therapy, assessment of Manual Muscle Testing.

	Right	Left
Elbow Flexors	5/5	5/5
Extensors	5/5	5/5
Wrist Flexors	5/5	2/5
Extensors	5/5	2/5

Diagnostic assessment

**Figure 1:**
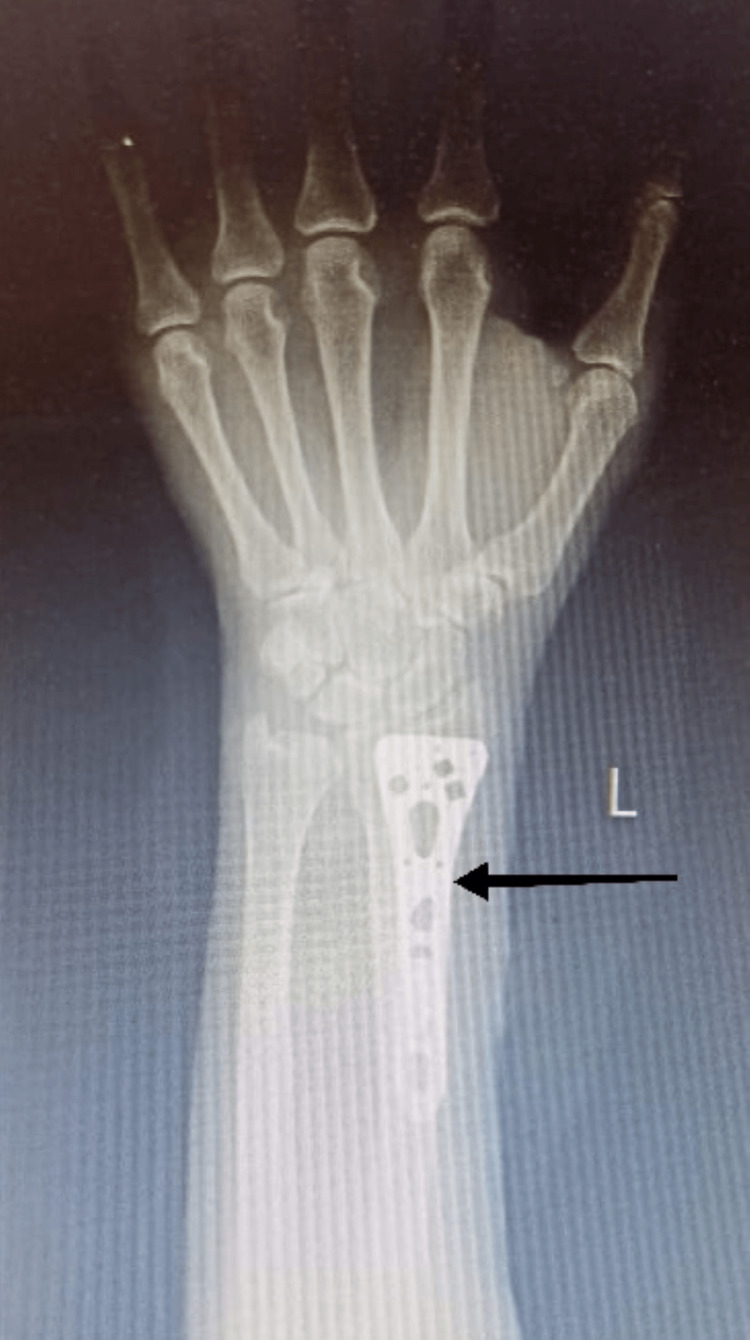
X-ray of patient’s left wrist postoperatively.

A 52-year-old female had road traffic accident and after x-ray (Figure 1) was diagnosed with volar Barton fracture left side without neurological deficits.

Timeline

On January 14, 2023, the patient was brought into the female orthopedic ward and after necessary investigations the patient wad planned for surgery, so on January 16, 2023, the patient underwent for ORIF with plate osteosynthesis for distal end radius fracture left side. The patient was provided with a referral for physical therapy on the first following surgery day. The timeline from the date of admission to the date of discharge is provided in Table 3.

**Table 3:** Timeline of the patient.

Timeline	
Admission date	14/01/2023
Assessment date	15/01/2023
Surgery date	16/01/2023
Physical therapy rehabilitation date	17/01/2023
Discharge date	27/01/2023

Therapeutic intervention

Phase 1 (Day 1 - One Week)

First, the patient was informed regarding her condition and how physiotherapy would benefit her. The patient was given active range-of-motion exercises to boost the mobility of his shoulder, fingers, and thumb with the goal of avoiding any consequences. Elbow flexion and extension exercises were also given, as well as isometric exercises for the intrinsic hand muscles.

Phase 2 (Week 2 - Week 4)

All of the above exercises along with a ROM were given to both metacarpophalangeal and interphalangeal joints as the swelling began to decrease. Exercises for the wrist flexors and extensors in addition to isometric exercises for the intrinsic hand muscles were initiated.

Phase 3 (Week 4 - Week 6)

Open chain kinetic exercises were given to enhance range of radial-ulnar deviation, pronation and supination motions (like scooping objects and dumping it in a box) and for improving writing skills and power grip ulnar deviation exercises were emphasized. To build muscle strength, gentle resistance exercises were prescribed, such as squeezing a ball.

Phase 4 (Week 6 - Week 8)

Active-assisted and passive ROM exercises were carried out to increase her wrist's ROM as the joint was slightly stiff. The patient continued the gentle resistive exercise by applying resistance with her unaffected extremity. For enhancing blood flow to the wrist joint and reducing swelling, an ice pack was applied daily for about 10 minutes till the swelling was reduced.

Phase 5 (Week 8 - Week 12)

**Figure 2:**
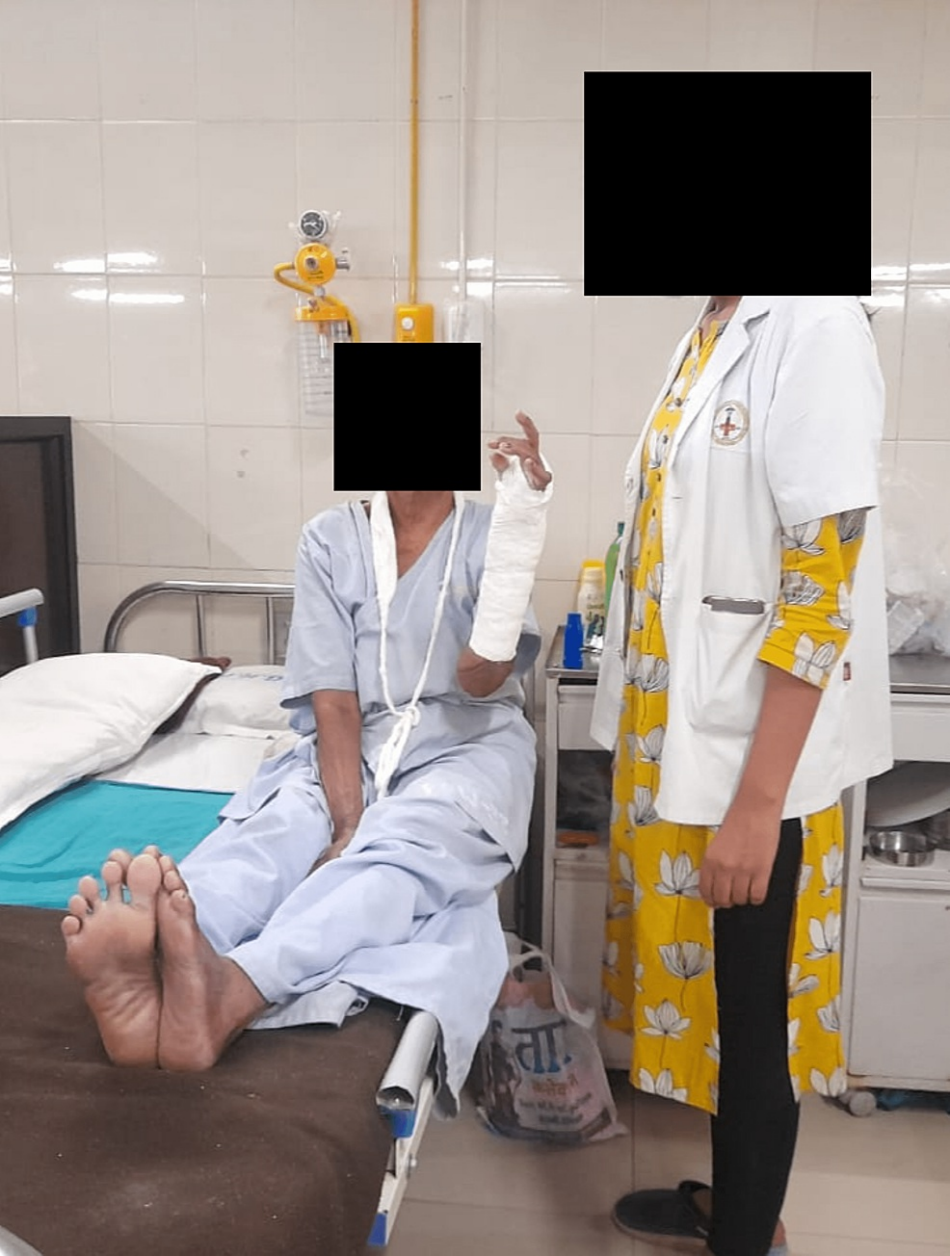
Finger opposition.

Along with the other exercises listed above, the patient was also instructed to practice stress supination and ulnar deviation exercises to enhance her daily living activities. To build up the muscles, weighted resistive exercises were done gradually. Figure 2 shows patient performing finger opposition.

Follow-up and outcomes

For three months, the patient made regular visits to the musculoskeletal OPD. After three months, no complaints were brought up by the patient in a subsequent physical assessment. The patient claimed to have a complete ROM at three months' time, with only minimal pain. Tables 4, 5 show ROM and manual muscle testing results on last day of rehabilitation, respectively.

**Table 4:** On the last day of rehabilitation, the range of motion of left-side joint assessed.

Joint	Active Range of Motion (in degree)	Passive Range of Motion (in degree)
Elbow Flexion	0-148	0-150
Forearm Pronation	0-78	0-80
Supination	0-76	0-80
Wrist Flexion	0-72	0-75
Extension	0-55	0-60
Radial deviation	0-12	0-15
Ulnar deviation	0-25	0-28

**Table 5:** On the last day of physical therapy, assessment of Manual Muscle Testing.

	Right	Left
Elbow Flexors	5/5	5/5
Extensors	5/5	5/5
Wrist Flexors	5/5	4/5
Extensors	5/5	4/5

The Upper Extremity Functional Index was used to measure the pre and post rehabilitation outcomes. This scale contains 20 items with maximum score of 80, which indicates highest functional status and 0 as lowest. Table 6 shows pre and post UEFI results.

**Table 6:** Finding of the Upper Extremity Functional Index prior to and after rehabilitation.

	Pre-rehabilitation	Post-rehabilitation
UEFI Score	45	75

## Discussion

As suggested by Ibrahim et al., fractures of the distal radius contribute to one-sixth of the total fractures encountered in the emergency room. These types of injuries tend to occur in the elderly community. When it comes to distal radius fractures, these are limited to occurring up to 3 cm from the radiocarpal joint and only in that area. There are a number of therapeutic methods accessible, like external fixation, closed reduction, plaster application, ORIF, and more. It's important to get the best treatment to provide great anatomical reduction and healing of fracture fragments so as to avoid long-term problems [10]. According to Skouras et al., in most cases, non-displaced fractures are treated non-operatively with a plaster cast. Displaced fractures may be non-operatively managed when they have a stable reduction. Radial fractures that are displaced or unstable are operated on. The choice may also be influenced by the fracture type, which may be intra-articular or extra-articular in addition to stability and displacement [11]. As reported by Tang et al., there are 1.2%-4.2% of distal radial fractures that are volar Barton fractures, which may be caused by high- or low-energy trauma. In several cases of severely comminuted fracture or osteoporosis, functional exercises are advised two to three weeks following surgery. For volar Barton fractures to have a positive prognosis, it is crucial to have a strong reduction, a firm internal fixation, and the right function exercises [12]. Szymanski et al. found that achieving a wrist joint that can move freely without experiencing any discomfort is the core goal of rehabilitation. Rehabilitation and physical therapy are continued during three stages of treatment: splinting, mobilization, and endurance training. Even when wearing a splint, complete finger, elbow, and flexion of the shoulder are advisable to prevent stiffness. Three to six weeks typically are needed for immobilization [13]. In this case, we saw the use of various therapeutic approaches like passive movements, isometric exercises, active assistive movements, open chain kinetic exercises, and progressive resistive exercises which led to achieving a fully functional range of the knee, improvement in the strength of muscle in a span of three months after starting physiotherapy rehabilitation.

## Conclusions

The Volar Barton fracture is a challenging condition to deal with and has several secondary consequences. Physiotherapy plays a key role in the rehabilitation and care of individuals with Volar Barton fractures. Early treatment of a volar Barton fracture may prevent prolonged tests, therapeutic delays, and undesirable long-term effects. In the medical-surgical treatment of volar Barton fractures, physiotherapy is essential. During the mobilization period, objectives of pain and edema control, in addition to enhanced wrist mobility, are maintained. Prolonged wrist stiffness as well as recurrent visits to therapy are consequences of delayed immobilization. Physiotherapeutic treatments like cryotherapy, ROM exercises, and exercises to strengthen the muscles can be given to individuals suffering from volar Barton fractures.
